# Temperament and Sense of Coherence: Emotional Intelligence as a Mediator

**DOI:** 10.3390/ijerph17010219

**Published:** 2019-12-27

**Authors:** Małgorzata Szcześniak, Klaudia Strochalska

**Affiliations:** Institute of Psychology, University of Szczecin, 71-017 Szczecin, Poland; klaudiastrochalska@tlen.pl

**Keywords:** temperament, sense of coherence, emotional intelligence

## Abstract

Sense of coherence (SOC) reflects an individual’s capacity and available resources to deal with stressful situations. For some time now studies have revealed that people differ in their experience of SOC; yet, very little is known about how and through which mechanisms a high level of SOC is formed. In order to increase our understanding about the paths to a high SOC in the stage of adulthood, we focused on exploring the role both of temperament, as it has been confirmed as a potential component in the development of more complex traits that emerge later in life, and of emotional intelligence (EI) as it has been found to increase SOC. The sample consisted of 173 participants between 18 and 49. We used the Temperament Evaluation of Memphis, Pisa and San Diego Autoquestionnaire (TEMPS-A), Orientation to Life Questionnaire (OLQ), and Emotional Intelligence Questionnaire (INTE). Results showed a negative correlation between the depressive, cyclothymic, irritable, and anxious dimensions of temperament and SOC, and EI. There was also a positive correlation between hyperthymic temperament and SOC, and EI. EI correlated positively with a general sense of coherence and its three dimensions. The PROCESS macro for SPSS showed that emotional intelligence mediates the relationship between depressive, cyclothymic, hyperthymic, irritable and anxious temperament, comprehensibility, manageability, meaningfulness, and global orientation to life. On the basis of the obtained outcomes, it can be stated that emotional intelligence mediates the relationship between dimensions of temperament and dimensions of SOC.

## 1. Introduction

The sense of coherence (SOC) construct was introduced by Antonovsky [1] and defined as a global life orientation that perceives the internal and external world as comprehensible, manageable, and meaningful. SOC reflects an individual’s capacity and available resources to deal with stressful situations [2]. For some time now studies have revealed [3,4] that people differ in their experience of SOC. Systematic review has indicated that neuroticism inversely predicts SOC, while extraversion, conscientiousness, and agreeableness are its positive predictors [5]. Likewise, a diffuse/avoidant style reduces SOC as well as its components: comprehensibility and meaningfulness [6]. Moreover, SOC is influenced by resistance resources [7], processes of identity development [8], and family background [9]. Yet, very little is known about how and through which mechanisms a high level of SOC is formed [9,10]. In order to increase our understanding about the paths to a high SOC in the stage of adulthood, we focused on exploring the role both of temperament, as it has been confirmed as a potential component in the development of more complex traits that emerge later in life [11], and of emotional intelligence (EI) as it has been found to increase SOC [12].

Temperament has been defined as a set of individual differences in reactivity and self-regulation, affected by heredity, maturity, and experience [13]. Conceptual and empirical work highlight that temperament traits regulate numerous areas of everyday functioning [14]. For example, the sociability and activity subscales of temperament are positively correlated with the sense of coherence construct, while impulsive sensation-seeking, neuroticism–anxiety, and aggression–hostility are negatively associated with SOC [15]. Negative significant correlations are also found between the degree of depressiveness and SOC [16].

The EI construct has been broadly conceptualized as an individual manifestation of adaptive emotional functioning that encompasses the ability to perceive, comprehend, and regulate emotions in the self and in others [17,18,19]. Higher levels of EI, understood as a trait and as an ability, have been found to be associated with indices of well-being [19,20,21] and life satisfaction [22,23,24], as well as higher scores for empathy [25], subjective happiness [22], psychological resilience [26], self-control [27], self-efficacy [28], and sociability [27].

Despite the many studies conducted with respect to temperament, not much attention has been paid to its role in the development of SOC. Although, Fried et al. [29] revealed a negative correlation between a difficult temperament and SOC. Moreover, a temperamental predisposition to posttraumatic stress disorder (PTSD) constituted a moderator variable of the relationship between SOC and PTSD [30]. Hutchinson and colleagues [31] found negative correlations between SOC and temperamental subscales of neuroticism–anxiety and aggression–hostility, and positive correlations between SOC and activity, and sociability. With regard to temperament and EI, Gardner et al. [32] observed moderate correlations between the trait of EI and three dimensions of temperament (effortful control, extraversion, and orienting sensitivity). Their findings also suggested that EI is largely a biological-based tendency as the overall model was significant for EI and more than 40% of the variance was accounted for by temperament. Finally, the intensity of an individual’s SOC may be determined [33] both by their genetic strengths (temperament) and emotional resources (emotional intelligence). Based on the literature review presented so far, we hypothesized that:

**Hypothesis** **1** **(H1).** *Depressive, cyclothymic, irritable, and anxious dimensions of temperament correlate negatively with SOC and EI, while hyperthymic temperament correlates positively with SOC and EI.*

**Hypothesis** **2** **(H2).** *EI correlates positively with SOC.*

**Hypothesis** **3** **(H3).** *EI mediates the effect of five dimensions of temperament on global SOC and its dimensions (comprehensibility, manageability, and meaningfulness).*

Since our study is a cross-sectional design, it is crucial to provide a convincing rationale for the specified directions of causal effects [34,35] between temperament and SOC. In regard to temporal precedence, there is both theory-driven and data-driven evidence that SOC may be influenced by temperament. According to Antonovsky’s theory [1,2], although SOC originates from early childhood, it only becomes and remains relatively stable throughout adult life after the age of 30. Moreover, there is a general consensus that temperament has a broad impact on individual experience and interactions [36]. The regulative theory of temperament developed by Strelau [37] highlights the role of temperament in the development of personality and in the regulation of people’s relations with their environment. Empirical data [38] have shown that symptoms of anxiety and depression explain a great part of SOC variance in nonclinical and clinical samples.

## 2. Materials and Methods

### 2.1. Participants

The sample consisted of 173 participants between 18 and 49 (88% women). The average age was approx. 24 (M = 23.62; SD = 5.25). The biggest group was formed by studying respondents (61%), followed by studying and working (24%), and working (15%). In terms of place of residence, 24% of participants declared living in rural areas, 16% in a town with fewer than 50,000 inhabitants, 27% between 51,000 and 499,000, and 33% in a city with over 500,000 inhabitants.

### 2.2. Data Collection

The participants in the present study were recruited through Internet platforms, such as Facebook and Google. A general description of the research project included a link to the anonymous online survey. Prior to filling in the questionnaires, all of the respondents interested in taking part in the study were prompted with a web-based informed consent. Only after giving their agreement, the participants had access to the battery of questionnaires. They were not offered any rewards for participating. Respondents under the age of 18 were excluded from participation. The protocol was approved by the Bioethics Committee of the Institute of Psychology at the University of Szczecin and was conducted in accordance with the Declaration of Helsinki.

### 2.3. Assessment of the Temperament Evaluation of Memphis, Pisa and San Diego Autoquestionnaire (TEMPS-A)

The Temperament Evaluation of Memphis, Pisa and San Diego Autoquestionnaire (TEMPS-A) is a self-report measure designed to assess temperament in healthy subjects and psychiatric patients through the depressive, cyclothymic, hyperthymic, irritable, and anxious subscales [39,40]. Individuals with a depressive temperament tend toward rigid thinking, self-accusation, and shyness. Cyclothymic temperament refers to mood lability, superficial thinking, and intense emotions. People with a hyperthymic temperament characterize themselves with the highest number of “positive” qualities: optimistic view of life, sociability, and self-assurance. An irritable temperament implies higher energy and anger, a lower level of empathy, and dissatisfaction. An anxious temperament implies a tendency to worry, ruminate, and continuous tension. In the women’s version, the questionnaire contains 110 items (109 in the version for men) and shows substantial internal consistency of the scales. The TEMPS-A has a yes/no response format, and 20 items per scale. In the present study, the Cronbach alpha coefficients were as follows: total score (α = 0.91), depressive (α = 0.76), cyclothymic (α = 0.85), hyperthymic (α = 0.80), irritable (α = 0.84), and anxious (α = 0.88).

### 2.4. Assessment of the Orientation to Life Questionnaire (OLQ)

The Orientation to Life Questionnaire (OLQ), developed by Antonovsky [1], measures the sense of coherence and consists of three interrelated components. Comprehensibility refers to the degree to which individuals recognize internal and external stimuli as comprehensible, coherent, and clear [41]. Thus, this cognitive dimension of SOC helps in the management of stressful situations. Manageability implies the extent to which people dispose of resources that can be used to meet the necessities of the stimuli. It is the instrumental or behavioral dimension of SOC. Meaningfulness indicates the degree to which one believes that existence has meaning and that life challenges are opportunities and not only burdens. It is the motivational dimension of SOC. The Orientation to Life Questionnaire has 29 items and the responses are ranged on a seven-point Likert scale (from 1 = never to 7 = always). The higher SOC scores mean a stronger sense of coherence. Previous reported alpha coefficients varied between 0.85 and 0.91. In the current study, the Cronbach alpha coefficients were as follows: comprehensibility (α = 0.71), manageability (α = 0.79), meaningfulness (α = 0.80), and global orientation to life (α = 0.88).

### 2.5. Assessment of the Emotional Intelligence Questionnaire (INTE)

The Emotional Intelligence Questionnaire (INTE), a Polish version [42] of a questionnaire developed by Schutte et al. [17], is a well-known 33-item self-report measure of emotional intelligence. It is based on the Salovey and Mayer model: the appraisal and expression of emotion, the regulation of emotion, and the use of emotions in thinking and acting [43]. Respondents use a 5-point scale, where 1 = strongly disagree and 5 = strongly agree, to designate to what extent each item illustrates their agreement. The scores are from 33 to 165. The higher the score, the higher the EI. The internal consistency in the original studies [17] showed an alpha of 0.90. In the present study, the Cronbach alpha coefficient was α = 0.88.

### 2.6. Statistical Analysis

All statistical analyses were carried out using IBM SPSS Statistics software version 23 (IBM Corporation, Armonk, NY, USA). There were no missing values since the data were gathered through an Internet platform that did not allow respondents to proceed unless they answered all questions. Descriptive statistics of means, standard deviation, skewness, and kurtosis were computed with no violations of the normality assumption found on any of the variables.

The internal consistency (Cronbach’s α) was calculated for all instruments applied. The correlation analysis was done using Pearson’s *r* coefficient. Linear regression analyses were used to test the normality of residuals and constant variance (homoscedasticity), detect multicollinearity, and outliers, and examine if and how much sex, age, background, and place of residence, as potential confounders in the model, would distort the relationship between the exposure (temperament and its dimensions) and outcome (sense of coherence) variables.

**Figure 1:**
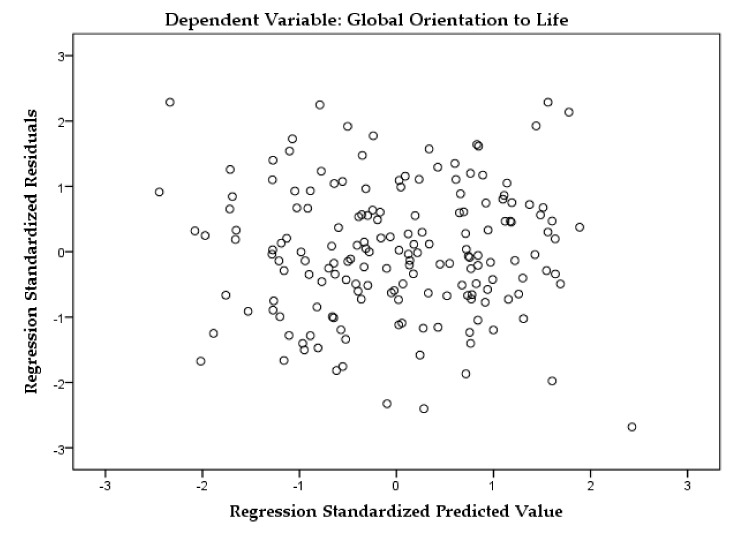
The scatterplot of the standardized residuals against standardized predicted values.

The PROCESS macro for SPSS (version 3.2, Heinrich-Heine-Universität, Düsseldorf, Germany) was run to establish the extent to which temperament influences sense of coherence through emotional intelligence. Temperament was the independent variable and the factor of SOC was the dependent variable. EI was included as a mediating variable. Thus, there were 20 single-level no. 4 mediation models [44], involving three-variable systems (Figure 1). For the present analysis, the 95% confidence interval of the indirect effects was calculated using 5000 bootstrapped resamples. This method appears to be superior to traditional mediation analyses because it does not require the data to adhere to assumptions of normality [45].

## 3. Results

### 3.1. Descriptive Statistics

The depressive, cyclothymic, hyperthymic, irritable, and anxious subscales of temperament, global SOC and its three dimensions, and global EI were screened for skewness and kurtosis to evaluate the normality of the scale’s distribution. We assumed values less than ±2 commonly considered acceptable as a normal distribution [45]. No variables exceeded the cutoffs of ±2 (Table 1).

### 3.2. Testing for the Normality of Residuals, Multicollinearity, and Confounders

In terms of the assumptions associated with the error term (residuals), homoscedasticity and autocorrelation were tested. A scatterplot of standardized residuals against the predicted values (Figure 1) shows that the plotted points had no obvious pattern nor systematic structure, being evenly divided above and below their mean value of zero [46]. Thus, the assumptions of normality, linearity, and homoscedasticity were met since the plotted values formed a rough rectangle.

The graphical presentation was confirmed by the Shapiro–Wilk test, which checks how closely residuals follow a normal distribution. The results of *p* = 0.95 for both unstandardized and standardized residuals suggested no deviations from normality. The Durbin–Watson statistic was 2.22, confirming that residuals were independent of each other, and indicating that the independence of the error terms was not a severe problem. In fact, values between 1.5 and 2.5 suggest that the assumption of non-autocorrelation was not violated [47].

Moreover, the regression model was tested to detect multicollinearity [48] because of correlations among all of the dimensions of temperament and the remaining variables. In the whole sample, the tolerance values ranged from 0.427 to 0.920, notably above the critical value of 0.1. The variance inflation factor (VIF) values ranged from 1.087 to 2.341 respectively, not exceeding the recommended threshold of 4.0 [49]. Both results suggest that multicollinearity was unlikely to be an issue in our study. The Mahalanobis distance procedure was evaluated, using Chi-squared distribution with a very conservative probability estimate for a case being an outlier (*p* < 0.001) [46]. Only four out of 173 cases were detected as possible multivariate outliers. Nonetheless, because the analyses run with and without the cases named as outliers showed that they did not have a large effect on the correlations, regressions, or mediations, and did not change the results [50], they were taken into consideration. With respect to age, sex, background, and place of residence as potentially confounding variables, they did not make a significant contribution to the model, as the change in the regression coefficient was very small between measurement with age (R^2^ = 0.689) and without (R^2^ = 0.674). However, in different equations and with different sets of independent or confounding variables, this outcome could potentially change.

### 3.3. Correlations between the Study Variables

In the next step, we verified the relationship between the dimensions of temperament, SOC, and EI (H1), and between SOC and EI (H2). Pearson’s *r* correlation (Table 2) showed a negative correlation between the depressive, cyclothymic, irritable, and anxious dimensions of temperament and SOC, and EI. Concomitantly, there was a positive correlation between hyperthymic temperament and SOC, and EI. Emotional intelligence correlated positively with a general sense of coherence and its three dimensions (H2).

### 3.4. Mediations

**Figure 2:**
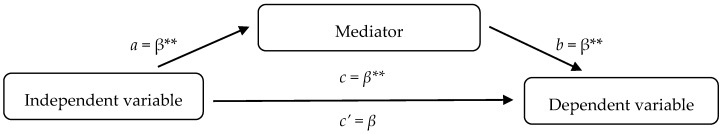
Theoretical model of the role of the mediator in the relationship between dimensions of temperament and dimensions of SOC; ** *p* < 0.01.

In the following part of the analyses, EI was introduced as a potential mediator which could weaken, strengthen or have no influence on the existing correlation between the independent variable (dimensions of temperament) and the dependent variable (dimensions of SOC) (Figure 2).

The PROCESS macro for SPSS showed (Table 3) that the c path (the direct effect) decreased after the introduction of EI and its dimensions in 20 out of 20 models (c’ path). On the basis of the obtained outcomes, it can be stated that emotional intelligence mediates the relationship between depressive, cyclothymic, hyperthymic, irritable, and anxious temperament, comprehensibility, manageability, meaningfulness, and global orientation to life.

## 4. Discussion

This research aimed at investigating the association between temperament, SOC, and EI (H1 and H2) as well as the possible mediatory effect of EI on the relationship between temperament and SOC (H3). Specifically, we expected a negative correlation between the depressive, cyclothymic, irritable, and anxious dimensions of temperament and SOC, and EI. We also assumed a positive correlation between hyperthymic temperament and SOC, and EI. Concurrently, we posited that EI would correlate positively with a general sense of coherence and its three dimensions, and would mediate the relationship between depressive, cyclothymic, hyperthymic, irritable and anxious temperament, comprehensibility, manageability, meaningfulness, and global orientation to life. The outcomes supported our three hypotheses and are in line with previous research.

Firstly, a negative correlation between depressive temperament and SOC confirmed the results obtained by Blom and colleagues [38], in which they observed an inverse association between generalized symptoms of depression and sense of coherence both in nonclinical and clinical samples. In the aforementioned study, depression, measured with Beck’s Depression Inventory, was also the strongest predictor of SOC. Similarly, Kurowska and Ciesielska [51] found that the level of depression grows with a lower level of sense of coherence. In respect to EI, our results refer to earlier studies. For example, in a recent study [52], it was noticed that adolescents and adults who exhibit lower levels of depression report higher EI as well. Correspondingly, individuals who display a higher ability to discriminate among feelings and regulate emotional states show less depression [53].

Since a close relationship between depression and anxiety has been acknowledged by many researchers [54], it is comprehensible that anxious temperament is negatively correlated with SOC. It has been established that people suffering from anxiety disorders have a lower level of sense of coherence [51]. Both before and after adjustment for potential confounders, a significant negative association between the presence of anxiety and SOC was observed [55]. Likewise, a longitudinal study by Carmel and Bernstein [56] showed strong and significant correlations between the A-trait and sense of coherence in three different measurements, although the associations became weaker over time. In terms of anxiety and EI, it is well documented that individuals with high self-reported emotional intelligence better tend to regulate the negative affect [53]. In the first reported investigation of the relationship between indices of social and performance anxiety and EI [57], the authors found high negative correlations between both types of anxious behavior and emotional intelligence.

Our study also showed positive correlations between hyperthymic temperament (characterized by optimism, sociability, self-esteem, resilience), SOC, and EI. The results confirm previous findings in which the propensity to perceive a greater sense of one’s own value played a positive role in the sense of coherence [58,59] and in EI, known as the ability to notice, understand, and manage emotions in oneself and others [60]. Moreover, since SOC is an inclusive concept [61] and has much in common with resilience, our results reflect the outcomes of Kesebir et al. [62] who observed a strong relationship between hyperthymic temperament and resilience both in depressive and healthy individuals.

With respect to a negative correlation between a cyclothymic temperament and SOC, there is some evidence [63] that mood lability seems to affect the development of a cohesive sense of self. In other studies [64], in the general population sample, two different measures of affective lability correlated negatively with identity integrity and the SOC total score, and comprehensibility, manageability, and meaningfulness subscales. Furthermore, affective instability was negatively associated with clarity of emotion, which refers to how clearly one recognizes and discriminates among one’s own emotions [65]. Since irritable temperament is conceptually linked to cyclothymic temperament [66], its negative correlation with SOC and EI is fully justified.

Finally, as we expected, H3 found its confirmation since in 20 out of 20 models, emotional intelligence acted as a mediator in the relationship between different dimensions of temperaments and the sense of coherence. These results are consistent with earlier studies [67] that reported a mediatory role of EI between some personality traits and positive resources. Such a mediatory effect may be because EI allows monitoring and management of one’s own emotions to guide one’s thinking and actions [68]. Previous researchers [69] have shown that EI mediated the relationship between emotional instability and happiness. Such outcomes have confirmed that awareness of internal sources of dissatisfaction might help people to overcome less functional tendencies that contribute to unhappiness. By analogy, successful affective information processing and management of emotions in the context of typical features of different types of temperaments might facilitate individuals in finding a sense of coherence in their life experiences.

## 5. Limitations

Our findings highlight the mediatory influence of emotional intelligence on the relationship between temperament and sense of coherence. However, a few limitations are important to note. The first limitation is that the cross-sectional nature of the data does not allow for the establishment of causal associations among the variables. Longitudinal and experimental studies would provide a more accurate representation of the relationships examined. Secondly, because of the unequal ratio of female to male respondents in this study, a cautious interpretation is suggested when applying the outcomes beyond the gender difference. We recommend that in future research, the same study should be performed with a greater participation of men. Thirdly, the sample was constrained to Internet users who could be unrepresentative of the population. Hence, researchers should investigate more diverse online groups. Fourthly, since the data were gathered through self-reports, they could be affected by the tendency to give responses that make the participant look good (socially desirable responding; SDR) [70]. Although the respondents were assured of anonymity, which is a common practice that allows bias to be reduced [71], the results obtained in our study could be affected by two response styles that include claiming socially desirable traits for the self (attribution responses) or disclaiming unwanted characteristics (denial responses) [72]. We suggest that in future studies, researchers concerned about social desirability bias might administer scales intended to screen each individual’s propensity toward SDR [70].

## 6. Conclusions

In terms of theoretical implications, the current research extends our insight into the interplay among temperament, EI, and SOC as the present study is one of few to have investigated this topic. Moreover, it provides meaningful evidence about the mediatory role of EI between temperament and SOC, shedding further light on how different types of temperament might affect global life orientation. The outcomes confirm Antonovsky’s theory that individuals’ SOC is in large part influenced by their general resources (temperament) and their ability to perceive, comprehend, and regulate emotions in the self (emotional intelligence). With respect to practical implications, the current findings suggest the importance of soft skills training (EI) for dealing with less functional temperamental tendencies in order to develop a deeper, clearer, and more coherent meaning of internal and external challenges.

## Figures and Tables

**Table 1:** Descriptive statistics for the Assessment of the Temperament Evaluation of Memphis, Pisa and San Diego Autoquestionnaire (TEMPS-A), Orientation to Life Questionnaire (OLQ), and Emotional Intelligence Questionnaire (INTE) (*n* = 173).

Scales	M	SD	Skewness	Kurtosis	α
1. DE	0.46	0.19	0.28	−0.18	0.75
2. CY	0.47	0.24	0.06	−0.88	0.85
3. HY	0.45	0.20	0.01	−0.44	0.78
4. IR	0.31	0.21	0.62	−0.31	0.82
5. AN	0.42	0.23	0.14	−0.78	0.86
6. CO	32.63	8.55	0.48	0.64	0.71
7. MA	50.29	9.60	0.01	−0.60	0.79
8. ME	38.02	9.33	−0.36	−0.30	0.80
9. OL	121.22	22.51	0.03	−0.29	0.88
10. EI	123.03	16.82	−0.59	0.68	0.88

Abbreviations: depressive temperament (DE); cyclothymic temperament (CY); hyperthymic temperament (HY); irritable temperament (IR); anxious temperament (AN); comprehensibility (CO); manageability (MA); meaningfulness (ME); global orientation to life (OL); emotional intelligence (EI).

**Table 2:** Correlation between dimensions of temperament, dimensions of sense of coherence (SOC), and EI (N = 173).

Dimensions of Temperament	CO	MA	ME	OL	EI
Depressive	−0.43 ***	−0.64 ***	−0.54 ***	−0.66 ***	−0.32 ***
Cyclothymic	−0.49 ***	−0.56 ***	−0.46 ***	−0.62 ***	−0.29 ***
Hyperthymic	0.34 ***	0.41 ***	0.53 ***	0.53 ***	0.39 ***
Irritable	−0.33 ***	−0.54 ***	−0.40 ***	−0.52 ***	−0.33 ***
Anxious	−0.50 ***	−0.58 ***	−0.31 ***	−0.57 ***	−0.19 *
EI	0.30 ***	0.39 ***	0.53 ***	0.50 ***	—

* *p* < 0.05*;* *** *p* < 0.001; comprehensibility (CO); manageability (MA); meaningfulness (ME); global orientation to life (OL); emotional intelligence (EI).

**Table 3:** The role of emotional intelligence in the relationship between dimensions of temperament and sense of coherence.

	a Path	b Path	c Path	c’ Path	Indirect Effect	B(SE)	Lower CI	Upper CI
1. DE–EI–CO	−28.18 ***	0.42 ***	−76.95 ***	−64.83 ***	−12.1160	4.2749	−21.3393	−4.9238
2. DE–EI–MA	−28.18 ***	0.11 ***	−31.99 ***	−28.71 ***	−3.2824	1.2654	−5.9397	−1.0964
3. DE–EI–ME	−28.18 ***	0.22 ***	−25.93 ***	−19.70 ***	−6.2371	2.3791	−11.6542	−2.3373
4. DE–EI–OL	−28.18 ***	0.42 ***	−76.95 ***	−64.83 ***	−12.1160	4.2951	−21.6365	−4.8290
5. CY–EI–CO	−20.67 ***	0.08	−17.44 ***	−15.65 ***	−1.7905	0.9721	−3.9663	−0.2523
6. CY–EI–MA	−20.67 ***	0.14 ***	−22.21 ***	−19.29 ***	−2.9270	1.0793	−5.2600	−1.1168
7. CY–EI–ME	−20.67 ***	0.23 ***	−17.88 ***	−12.92 ***	−4.9596	1.6969	−8.6323	−2.0151
8. CY–EI–OL	−20.67 ***	0.46 ***	−57.55 ***	−47.87 ***	−9.6771	3.3532	−17.1733	−4.0022
9. HY–EI–CO	32.45 ***	0.10 (ni)	14.39***	11.14 ***	3.2509	1.6395	0.1610	6.4865
10. HY–EI–MA	32.45 ***	0.15 ***	19.36***	14.31 ***	5.0453	1.6207	2.2367	8.5635
11. HY–EI–ME	32.45 ***	0.21 ***	24.29***	17.43 ***	6.8571	2.3151	3.0070	11.8999
12. HY–EI–OL	32.45 ***	0.51 ***	61.55***	42.22 ***	19.3345	4.0042	11.6369	27.2898
13. IR–EI–CO	−25.55 ***	0.10 **	−13.08 ***	−10.28 ***	−2.8029	1.3590	−6.0434	−0.6856
14. IR–EI–MA	−25.55 ***	0.13 ***	−23.89 ***	−20.40 ***	−3.4929	1.2400	−6.2208	−1.4522
15. IR–EI–ME	−25.55 ***	0.24 ***	−17.43 ***	−11.10 ***	−6.3284	2.0159	−10.7930	−2.9083
16. IR–EI–OL	−25.55 ***	0.49 ***	−54.42 ***	−41.79 ***	−12.6242	4.0136	−21.2732	−5.8575
17. AN–EI–CO	−14.23 **	0.10 **	−18.61 ***	−17.08 ***	−1.5261	0.9311	−3.6849	−0.0765
18. AN–EI–MA	−14.23 **	0.16 ***	−23.87 ***	−21.51 ***	−2.3586	1.1373	−4.6953	−0.2421
19. AN–EI–ME	−14.23 **	0.27 ***	−12.48 ***	−8.60 **	−3.8710	2.0551	−8.4428	−0.3687
20. AN–EI–OL	−14.23 **	0.54 ***	−54.97 ***	−47.21 ***	−7.7557	3.8812	−16.0240	−0.7991

** *p* < 0.01; *** *p* < 0.001; confidence interval (CI); nonsignificant (ni); depressive temperament (DE); cyclothymic temperament (CY); hyperthymic temperament (HY); irritable temperament (IR); anxious temperament (AN); comprehensibility (CO); manageability (MA); meaningfulness (ME); global orientation to life (OL); emotional intelligence (EI).
